# Unresectable primary hepatic adenosquamous carcinoma successfully treated with systemic and transcatheter hepatic arterial injection chemotherapies followed by conversion surgery: a case report and literature review

**DOI:** 10.1186/s12876-021-02070-3

**Published:** 2021-12-20

**Authors:** Yusuke Watanabe, Akihiko Osaki, Kiwamu Kimura, Shunta Yakubo, Kenichi Takaku, Munehiro Sato, Hideki Hashidate, Nobuo Waguri, Shuji Terai

**Affiliations:** 1 Department of Gastroenterology and Hepatology, Niigata City General Hospital, Niigata, Japan; 2 Division of Preemptive Medicine for Digestive Disease and Healthy Active Life, School of Medicine, Niigata University, 1-757 Asahimachi-dori, Chuo-ku, Niigata, 951-8510 Japan; 3 Division of Gastroenterology and Hepatology, Graduate School of Medical and Dental Sciences, Niigata University, Niigata, Japan; 4 Department of Pathology, Niigata City General Hospital, Niigata, Japan

**Keywords:** Adenosquamous carcinoma, Transcatheter hepatic arterial injection, Chemotherapy, Conversion surgery

## Abstract

**Background:**

Primary hepatic adenosquamous carcinoma (ASC) is a type of tumor that has the features of both adenocarcinoma and squamous cell carcinoma (SCC). The prognosis for patients with ASC is poor, as the chemotherapy has been ineffective so far.

**Case presentation:**

Here, we report a case of a 62-year-old male patient who presented with high fever. The tumor marker levels were high, and abdominal dynamic computed tomography showed a liver tumor and distant lymph node metastases. Upon further investigation, needle biopsy of the liver tumor showed a primary hepatic SCC. Because the SCC was unresectable, the patient was treated with tegafur/gimeracil/oteracil (S-1) and transcatheter hepatic arterial injection (TAI) of cisplatin. After chemotherapy, a surgical resection performed on the remaining liver tumor, made the patient cancer-free. After the operation, the liver tumor was confirmed as primary hepatic ASC. Subsequently, the patient was administered postoperative adjuvant chemotherapy, which prevented its recurrence.

**Conclusions:**

Due to the lack of an effective treatment for primary hepatic ASC, its prognosis is poor. Here, we suggest that a chemotherapy combination of 5-fluorouracil (S-1) and cisplatin along with conversion surgery might be an effective way for treating primary hepatic ASC. Our experience from this case shall be valuable to clinicians around the world involved in the treatment of primary hepatic ASC.

## Background

Adenosquamous carcinoma (ASC) is a tumor that has the features of both adenocarcinoma and squamous cell carcinoma (SCC) [1, 2]. Primary hepatic ASC, a variant type of cholangiocarcinoma, is a rare disease [3, 4]. Some of the primary hepatic ASC patients have been treated with surgery; however, no effective treatment including a systemic chemotherapy has been reported for unresectable cases, leading to a poor prognosis [4–8].

Here, we report the case of a patient with unresectable primary hepatic ASC, who has treated successfully with a combination of systemic and transcatheter hepatic arterial injection (TAI) chemotherapies, followed by a conversion surgery. In addition, we present a comprehensive comparative analysis of the treatment strategies of 72 cases of primary hepatic ASC (previously reported and our case), which will be valuable for clinicians around the world in making informed decisions while treating similar patients.

## Case presentation

**Fig. 1:**
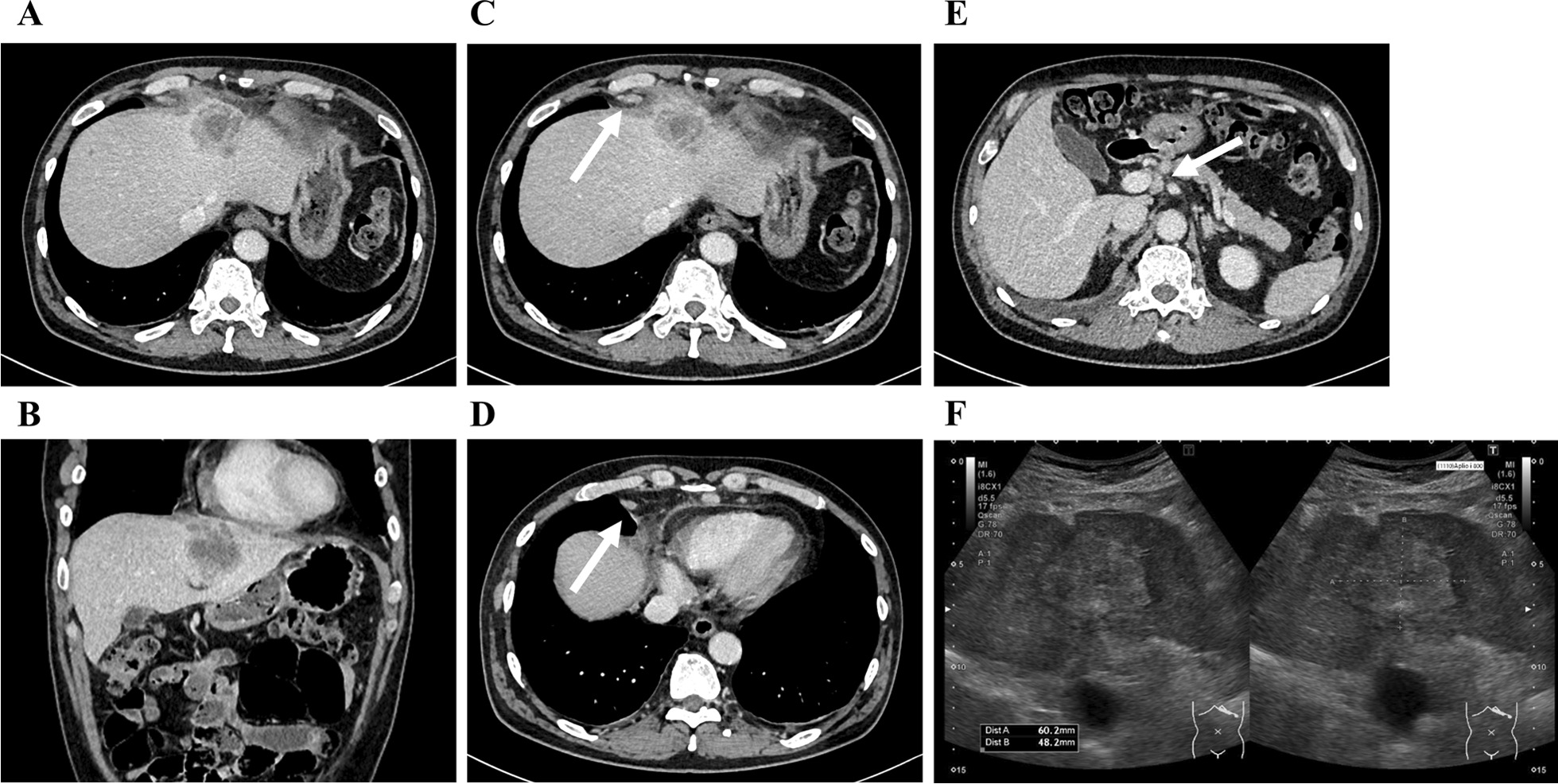
Abdominal contrast-enhanced computed tomography and ultrasonography on admission. **A**, **B** Abdominal contrast-enhanced computed tomography shows a tumor with a low-density area in the left lobe of the liver (**A** axial image, **B** sagittal image). **C**–**E** Axial images of the abdominal contrast-enhanced computed tomography shows swelling in multiple lymph nodes (white arrows). **F** Abdominal ultrasonography shows a hyperechoic solid tumor with 60.2 × 48.2 mm in diameter in the left lobe of the liver

**Fig. 2:**
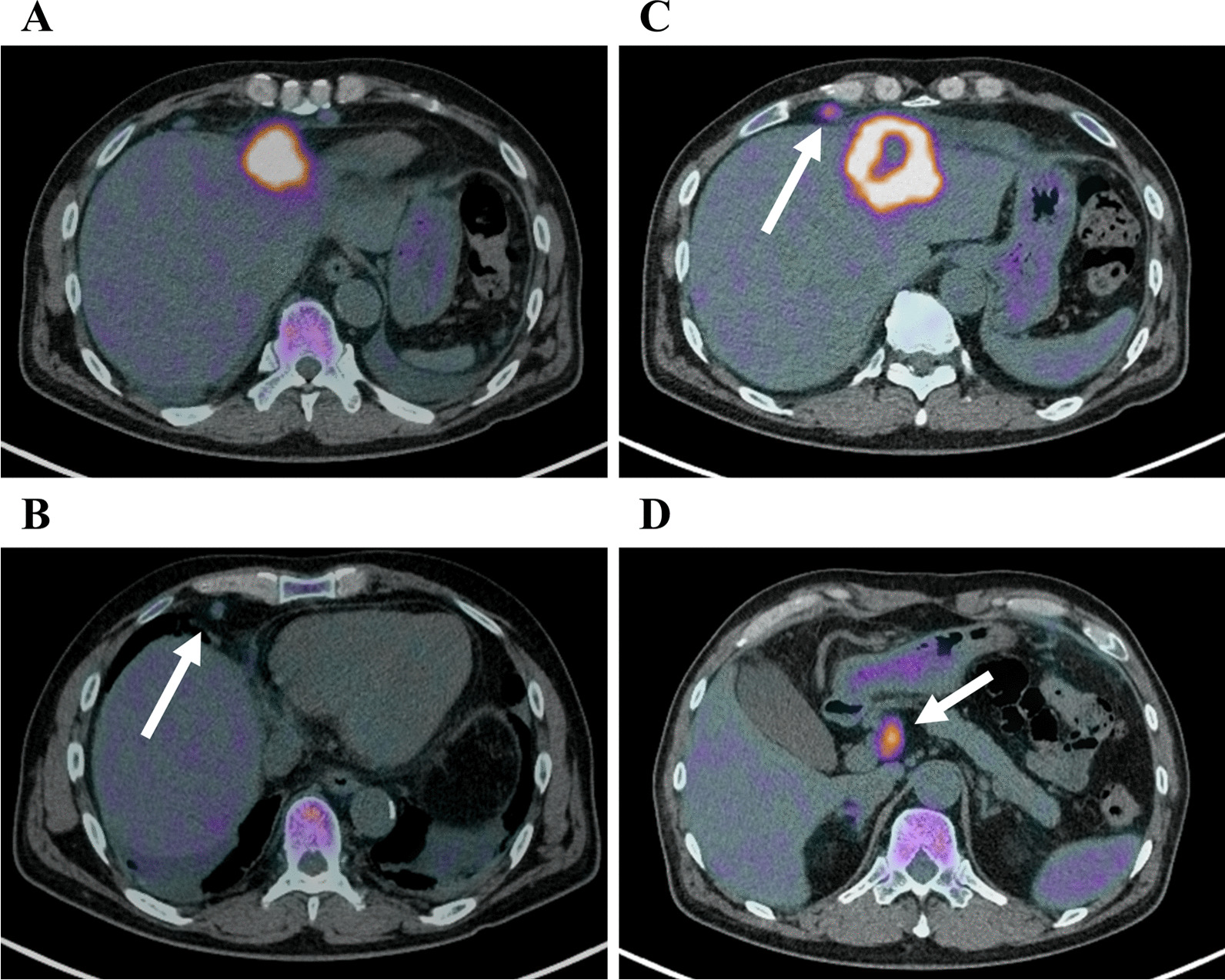
Abdominal fluorodeoxyglucose/positron emission tomography before systemic chemotherapy and transcatheter hepatic arterial injection. **A** Abdominal fluorodeoxyglucose/positron emission tomography shows a strong accumulation of fluorodeoxyglucose (FDG) in the tumor present in the left lobe of the liver. **B**, **C** Accumulation of FDG is observed in multiple lymph nodes around the heart (white arrows). **D** Accumulation of FDG is observed in the perihilar lymph node (white arrows)

**Fig. 3:**
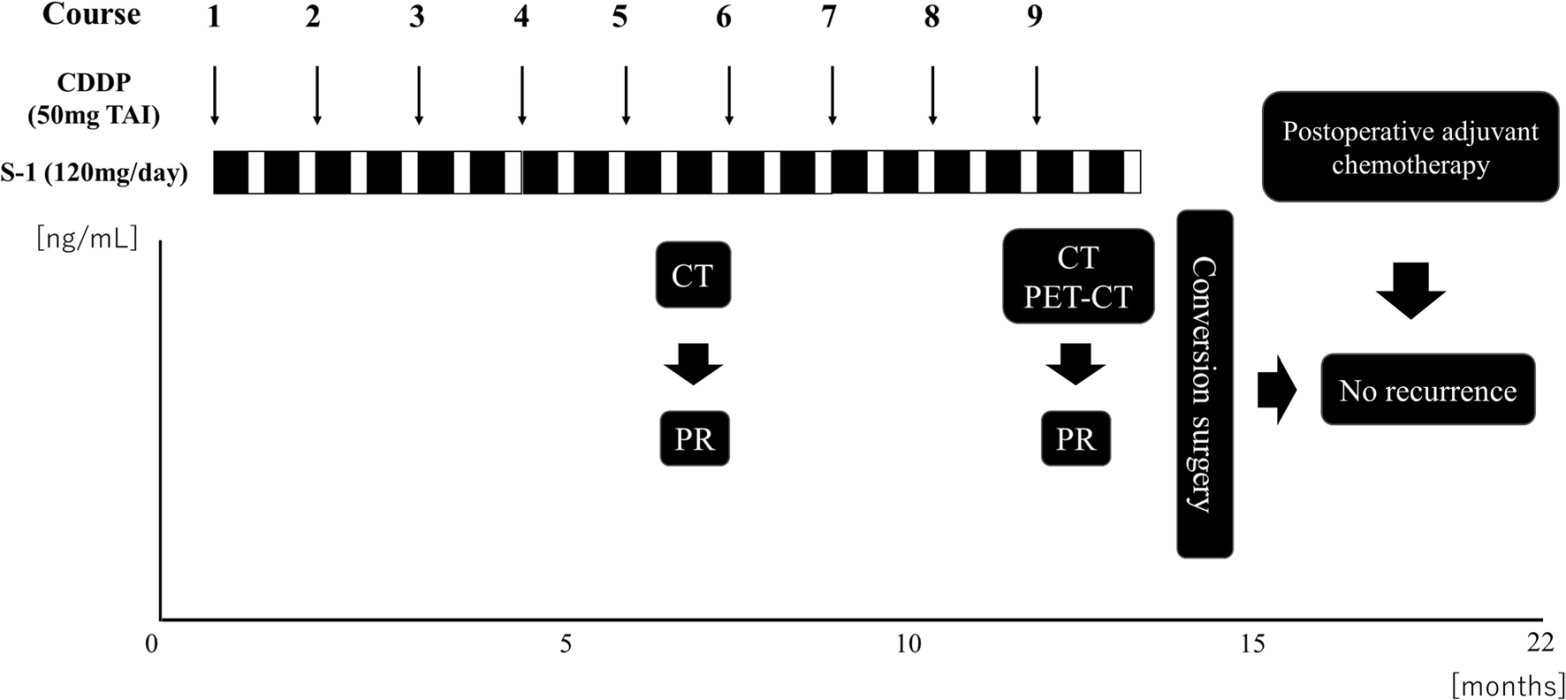
Changes in tumor markers during chemotherapy, conversion surgery, and postoperative adjuvant chemotherapy. Tumor markers gradually decrease along with systemic and transcatheter hepatic arterial injection chemotherapies. After 9 courses of treatment, the patient undergoes conversion surgery. Postoperative adjuvant chemotherapy was administered with no recurrence of the tumor. CDDP, cisplatin; CT, computed tomography; PR, partial remission; S-1, Tegafur/Gimeracil/Oteracil; TAI, transcatheter hepatic arterial injection; SCC, squamous cell carcinoma antigen; CYFRA, cytokeratin 19 fragment

**Fig. 4:**
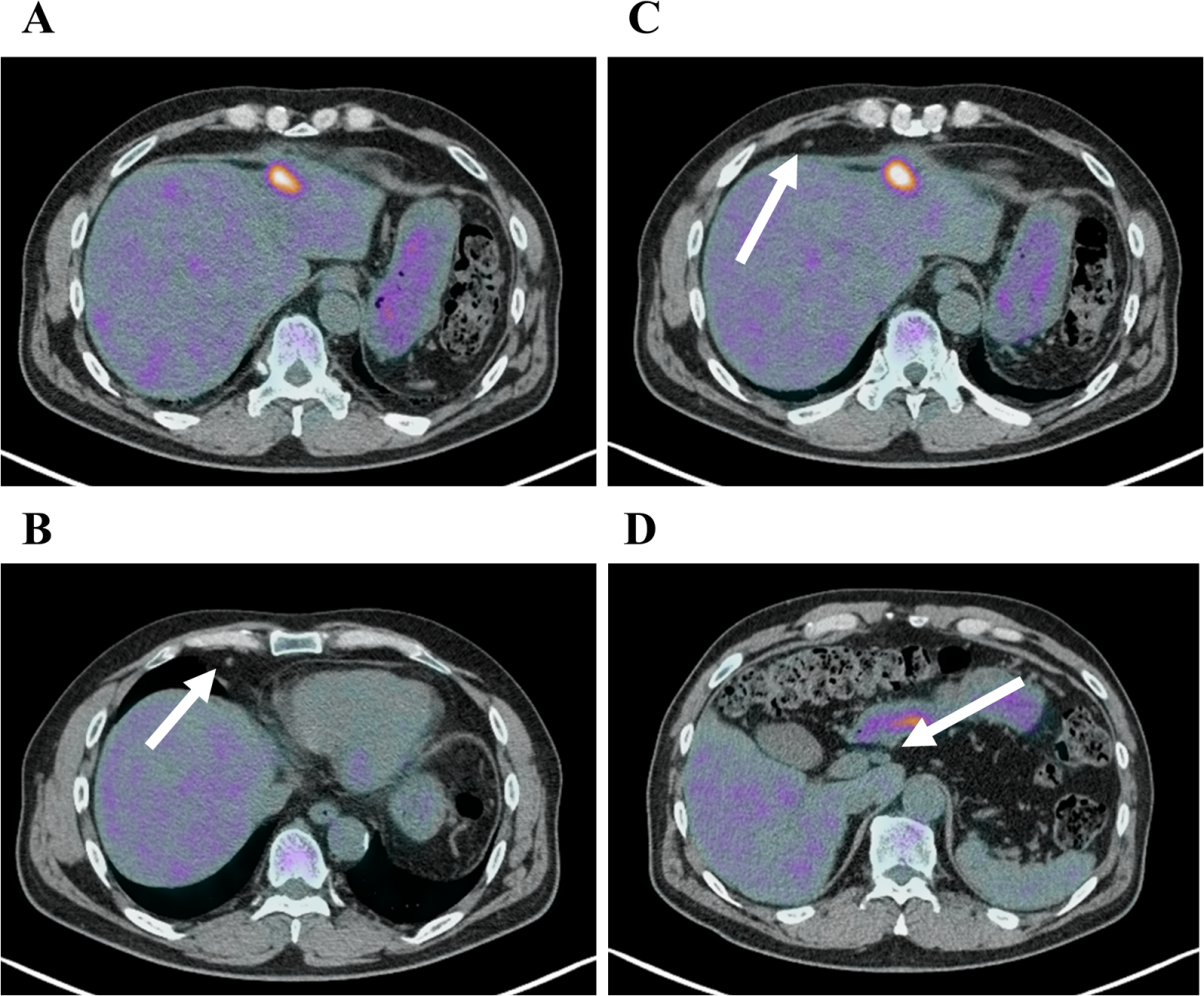
Abdominal fluorodeoxyglucose/positron emission tomography after chemotherapy and transcatheter hepatic arterial injection. **A** Accumulation of fluorodeoxyglucose (FDG) is observed in the primary tumor with reduced size after the treatment with systemic chemotherapy and transcatheter hepatic arterial injection. **B**–**D** Accumulation of FDG is not observed in the perihilar lymph node and multiple lymph nodes around the heart. The size of the lymph nodes is reduced after the treatment (white arrows)

**Fig. 5:**
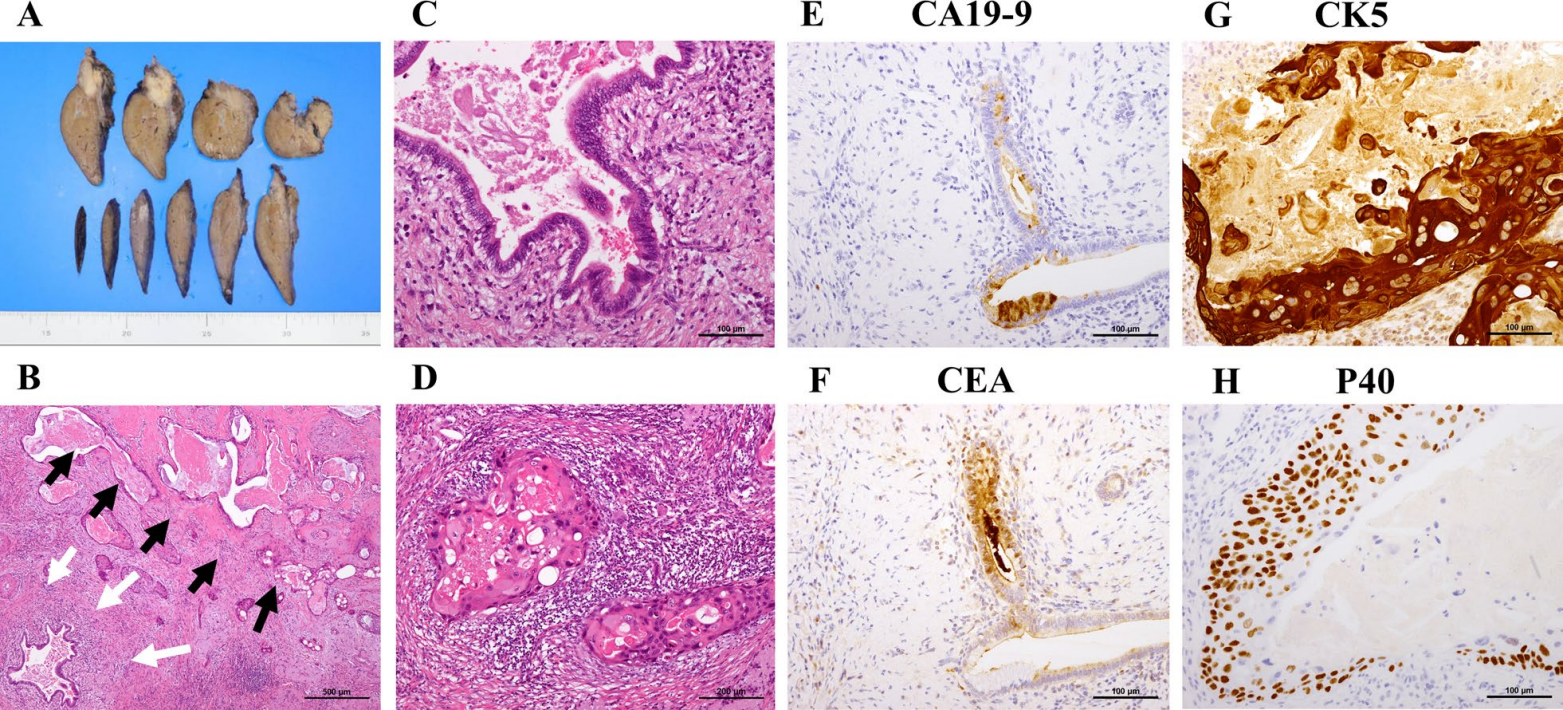
Pathological findings of primary tumor of the liver. **A** Whole mount bright-field images of a solitary nodule with 21 × 20 × 18 mm in diameter. **B** Hematoxylin eosin (HE) staining shows adenocarcinoma (white arrows) and squamous cell carcinoma (black arrows) component. **C** HE staining shows adenocarcinoma. **D** HE staining shows squamous cell carcinoma component with a keratinization. **E**, **F** Immunostaining of carbohydrate antigen (CA) 19-9, and carcinoembryonic antigen (CEA), shows positive expression in the adenocarcinoma region. **G**, **H** Immunostaining of cytokeratin (CK) 5, and p40 shows a positive expression in the squamous cell carcinoma region

A 62-year-old male patient with no adverse medical history was admitted to our hospital with a high fever. Contrast-enhanced computed tomography (CT) revealed a liver tumor in the left lobe of the liver (Fig. 1A–E). Due to the presence of high fever, elevated inflammatory markers, and detection of a low-density area in the liver tumor on CT, our initial diagnosis was liver abscess. However, the detection of elevated tumor marker levels and a clear imaging of a solid tumor on abdominal ultrasonography, confirmed our diagnosis of intrahepatic malignant tumor (Fig. 1F). Laboratory findings on admission are listed in Table 1. Further, a percutaneous ultrasound-guided needle biopsy of the liver tumor revealed it as SCC. The patient had no liver disease underlying the liver tumor. Endoscopic examinations showed that the upper and lower gastrointestinal tracts were free of primary malignancies. In addition, no head or neck malignancies were detected on CT. Fluorodeoxyglucose/positron emission tomography (FDG/PET) CT showed accumulation of FDG in the liver tumor, perihilar lymph nodes, and lymph nodes around the heart (Fig. 2A–D), These results confirmed our diagnosis of a primary tumor with multiple distant lymph node metastases. We suspected a diaphragmatic and pericardial invasion from the primary tumor as the tumor border was found to be obscured on CT. Therefore, we opted for systemic and TAI chemotherapies instead of surgery based on patient’s consent. The patient was administered with a TAI of 50 mg/body cisplatin (CDDP) (once every 6 weeks), for treating the primary tumor, and 120 mg/day tegafur/gimeracil/oteracil (S-1) systemically for both the primary tumor and distant metastases (repeating twice of 2-week administration and 1-week withdrawal). One course of chemotherapy was set at 6 weeks. A CT scan performed after five courses of chemotherapy, showed a reduction in the size of the primary tumor and lymph nodes. In addition, the tumor markers decreased after five courses of chemotherapy, which indicated partial remission (PR). Because no adverse events were reported from systemic and TAI chemotherapies after five courses, the treatment was continued for nine courses. A summary of treatments and changes in the tumor marker levels is shown in Fig. 3. After 9 courses of treatment, further reduction in the size of the primary tumor and lymph nodes was seen on CT scan. Additionally, FDG/PET CT showed no accumulation of FDG in the distant lymph nodes with a reduction in their size (Fig. 4A–D). Due to the reduction in the size of the tumor, the patient underwent conversion surgery, in which a laparotomic left lateral segmentectomy, and resection of the diaphragm and pericardium were performed, as the tumor invasion was observed (Fig. 5A–H). The patient also underwent sampling of perihilar lymph nodes, which showed no cancer cells. Finally, postoperative pathology tests performed on the resected tumor revealed that the patient was affected by primary hepatic ASC. Although the patient became cancer-free by conversion surgery, he was treated with adjuvant chemotherapy with intravenous CDDP (40 mg every 6 weeks) and S-1 (120 mg/day, repeating twice of 2-week administration and 1-week withdrawal) as adjuvant chemotherapy. No tumor recurrence was observed for 6 months postoperatively.

**Table 1:** Laboratory findings on the day of admission

Laboratory findings					
*Hematology*		*Biochemistry*		*Immunology*	
WBC	12,590/µL	AST	58 IU/L	HBsAg	(–)
neut	81.9%	ALT	51 IU/L	HBsAb	(–)
lym	12.2%	ALP	459 IU/L	HBcAb	(–)
eosino	0.7%	LDH	169 IU/L	HCVAb	(–)
mono	4.1%	γ-GTP	101 IU/L		
baso	0.2%	TP	7.2 g/dL		
RBC	404 × 10^4^/µL	Alb	2.9 g/dL		
Hb	11.9 g/dL	T-bil	0.6 mg/dL		
Hct	37.6%	D-bil	0.2 mg/dL		
Plt	57.5 × 10^4^/µL	CRP	15.8 mg/dL		
*Tumor marker*		BUN	11.7 mg/dL		
CEA	4 ng/mL	Cre	0.81 mg/dL		
CA19-9	2.1 U/mL	UA	4.1 mg/dL		
AFP	1.3 ng/mL	Na	138 mEq/L		
PIVKA-II	12.9 mAU/mL	K	4.3 mEq/L		
SCC	18.6 ng/mL	Cl	102 mEq/L		
CYFRA	5.01 ng/mL	PCT	0.06 ng/mL		

WBC, white blood cell; neut, neutrophil; lym, lymphocyte; eosino, eosinophil; mono, monocyte; baso, basophil; RBC, red blood cell; Hb, hemoglobin; Hct, hematocrit; Plt, platelet; CEA, carcinoembryonic antigen; CA19-9, carbohydrate antigen 19–9; AFP, alfa-fetoprotein; PIVKA-II, protein induced by vitamin K absence or antagonist-II; SCC, squamous cell carcinoma antigen; CYFRA, cytokeratin 19 fragment; AST, aspartate aminotransferase; ALT, alanine aminotransferase; ALP, alkaline phosphatase; LDH, lactate dehydrogenase; γ-GTP, gamma-glutamyl transpeptidase; TP, total protein; Alb, albumin; T-bil, total bilirubin; D-bil, direct bilirubin; CRP, C-reactive protein; BUN, blood urea nitrogen; Cre, creatinine; UA, uric acid; Na, sodium; K, potassium; Cl, chlorine; PCT, procalcitonin; HBsAg, hepatitis B virus surface antigenemia; HBsAb, hepatitis B virus surface antibody; HBcAb, hepatitis B virus core antibody; HCVAb, hepatitis C virus antibody

## Discussion and conclusions

ASC is a combination of adenocarcinoma and SCC with cellular keratinization and intercellular bridges in the tumorous tissue [1, 2]. Although there are a few cases of ASC reported in the gallbladder [9, 10], stomach [11, 12], and pancreas [13, 14], the incidence of hepatic ASC is extremely rare [4]. There are no identified risk factors for ASC. The disease is difficult to detect and progresses rapidly; therefore, by the time it is diagnosed, the disease has advanced significantly, making it difficult to treat [5]. Hence, the prognosis of primary hepatic ASC is poor as compared to the usual intrahepatic cholangiocarcinoma. The 1-year, 2-year, and 3-year survival rates of usual intrahepatic cholangiocarcinoma are 32.8%, 14.8%, and 9.5%, respectively, whereas the 1-year survival rate of primary hepatic ASC is 18.8%, and the survival rate after the second year is less than 1% [6, 8, 15].

The common symptoms of primary hepatic ASC are nonspecific, such as fever, loss of appetite, weight loss, malaise, and abdominal pain. Even in the advanced stages of primary hepatic ASC, there are cases with not so elevated tumor markers [16–18]. There are cases with elevated adenocarcinoma markers [19–21], or elevated SCC markers [2, 22]. To add to the complexity, the primary hepatic ASC has no specific signatures on ultrasonography and CT. Therefore, it is quite difficult to define the diagnostic criteria for primary hepatic ASC. Owing to tissue necrosis associated with primary hepatic ASC, it can be easily confused with the liver abscess in the imaging data. Therefore, tissue pathology should be conducted to confirm the diagnosis of primary hepatic ASC [1, 23–27]. In the present case, we suspected a liver abscess initially as the patient was febrile, had elevated inflammatory markers, and his CT scan showed a low-density area in the center of the tumor. However, imaging of a solid mass on the liver through abdominal ultrasonography and detection of elevated tumor markers led us to modify our diagnosis to intrahepatic malignant tumor. The risk of cancer dissemination associated with percutaneous liver puncture was considered acceptable as the patient already had distant lymph node metastases. Thus, a percutaneous needle biopsy of the liver tumor was performed, which further confirmed that the tumor was SCC. For a better assessment of the tumor, we performed FDG/PET CT, where FDG accumulated in the liver tumor and lymph node metastases. This procedure was useful for diagnosis and staging of the tumor.

Apart from surgery, there is no effective treatment reported so far for primary hepatic ASC. However, the prognosis is poor even after successful surgical resection and the postoperative recurrence rate of the ASC is high [7, 8]. With systemic chemotherapy or selective treatment into the liver such as TAI, the effective chemotherapy treatment regimens are unknown. In this case, the patient was administered S-1 systemically and CDDP as TAI. The chemotherapy treatment regimen used here was available for cholangiocarcinoma in our institution, and was described in previous reports [27–29]. Since intensive treatment for the prognostic lesions is a relatively common treatment strategy in patients with unresectable, the patient was administered CDDP through TAI. Although there was an option to perform surgery first, preoperative systemic and TAI chemotherapies contributed in reducing the size of the tumor, thus, the extent of resection and invasiveness of surgery was also reduced. Post-surgery, the patient successfully became cancer-free. Additionally, postoperative adjuvant chemotherapy was administered with similar regimen, which prevented tumor recurrence. Although S-1 and CDDP drugs were effective in this case, it was difficult to assess the efficacy of this regimen based on this one case alone. Therefore, we conducted a literature review of similar cases to analyze and identify the most efficacious treatment for primary hepatic ASC.

**Fig. 6:**
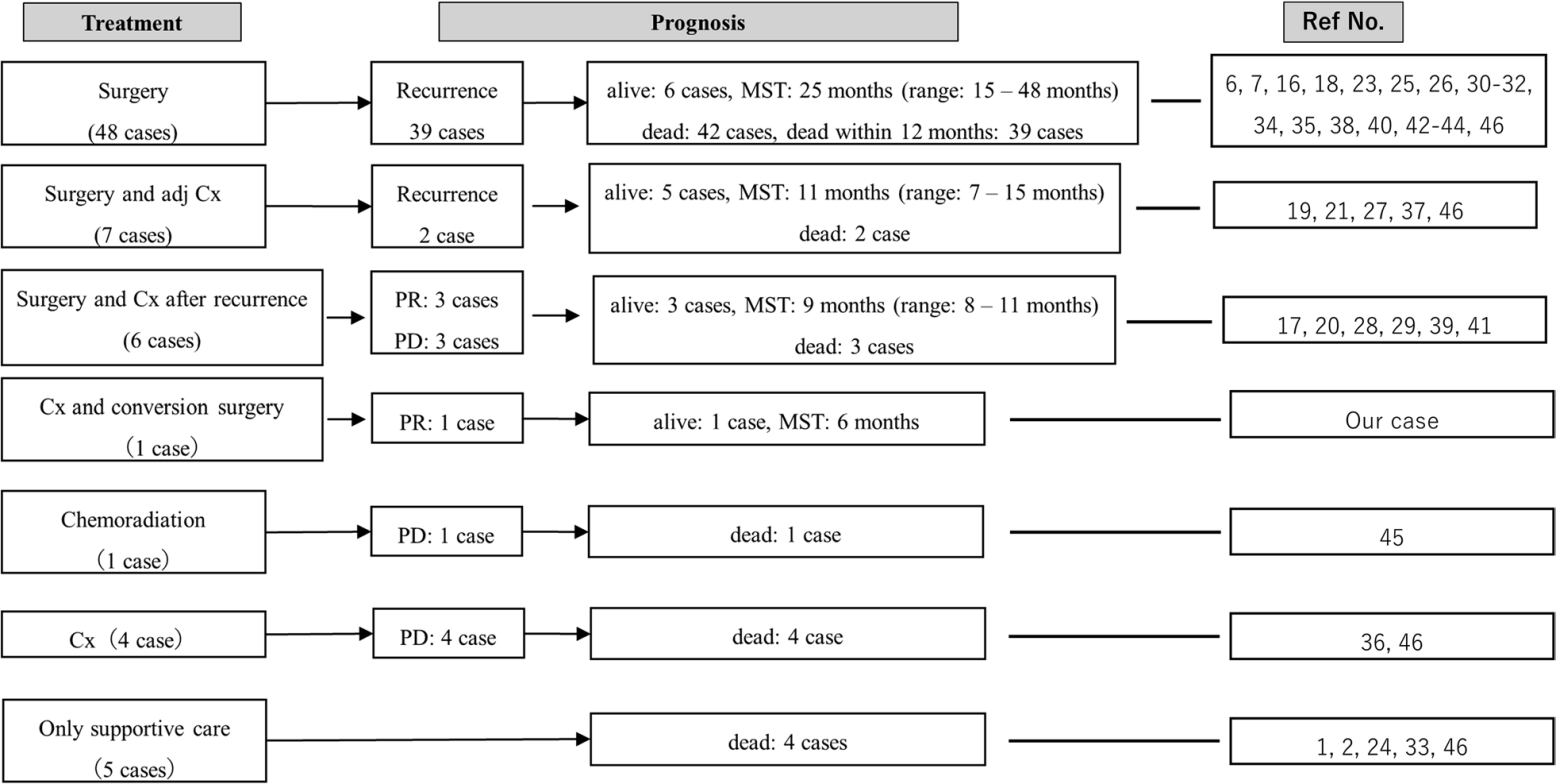
Summary of 72 cases with primary hepatic adesquamous carcinoma. The summary of 72 cases included surgery alone in 48 patients, surgery followed by postoperative adjuvant chemotherapy in seven patients, surgery and chemotherapy after recurrence in six patients, chemotherapy and conversion surgery in one patient, systemic chemotherapy alone in four patients, chemoradiation in one patient, and only supportive care in five patients. The clinical detail information of 19 patients in which chemotherapy was used for treatment (surgery followed by postoperative adjuvant chemotherapy, surgery and chemotherapy after recurrence, chemotherapy and conversion surgery, systemic chemotherapy alone, and chemoradiation) are summarized in Table 2. Cx, chemotherapy; PD, progressive disease; PR, partial remission; Ref No., reference number; y.o., years old; MST, median survival time

A literature search on PubMed using the terms “adenosquamous carcinoma” and “liver” retrieved 71 published cases on primary hepatic ASC. The available information on all the 72 cases (including our case) is summarized in Fig. 6 [1, 2, 6, 7, 16–21, 23–46]. The patients in previously reported cases included 42 males and 30 females, with ages 28 through 85 years (median age: 65 years). The treatment regimen followed in these patients included surgery alone in 48 patients, surgery followed by postoperative adjuvant chemotherapy in seven patients, surgery and chemotherapy (due to recurrence after surgery) in six patients, chemotherapy and conversion surgery in one patient (our case), systemic chemotherapy alone in four patients, chemoradiation in one patient, and only supportive care in five patients. In the surgery alone cases, postoperative recurrence was observed in 39 patients (81.2%), which indicates a very high recurrence rate. Postoperatively, 42 patients (85.1%) died, of which 39 patients (92.9%) died within 12 months. These results are consistent with the poor prognosis of primary hepatic ASC patients after surgery. The clinical information of 19 patients (surgery followed by postoperative adjuvant chemotherapy, surgery and chemotherapy after recurrence, chemotherapy and conversion surgery, systemic chemotherapy alone, and chemoradiation) treated with chemotherapy, including the details of the drugs used is summarized in Table 2 [17, 19–21, 27–29, 36, 37, 39, 41, 45, 46]. In patients who received postoperative adjuvant chemotherapy, tumor recurrence was prevented in five out of seven patients. This result clearly indicates that adjuvant chemotherapy may improve patient prognosis after surgery. The 12 patients who received non-adjuvant chemotherapy are difficult to compare as a different chemotherapy regimen was followed in each case. Four out of 12 patients became PR conditions, and three PR patients were treated with 5-fluorouracil (5-FU) (including S-1) and CDDP, and with combined TAI. One of these three PR patient is our case. Additionally, our patient is the only case who was successfully treated with systemic chemotherapy, TAI, and conversion surgery. These results suggest that chemotherapy with 5-FU (S-1) and CDDP in combination with TAI along with conversion surgery might be an effective strategy for treating primary hepatic ASC. However, future studies with further number of patients would be necessary to better understand the effectiveness of this combined treatment strategy. In addition, this analysis is only a case report and a literature review based on published cases, which has inherent limitations, such as lack of ability to generalize, no possibility to establish cause-effect relationship, potential for overinterpretation, and publication bias.

**Table 2:** Summary of 19 cases in which chemotherapy was used for treatment

No.	Regimen	Object	Administration route	Response	Prognosis	References
1	5-FU + CDDP	Adj	Intravenously	NR	Alive, 8 months after surgery	[27]
2	GEM + S-1	Adj	Intravenously	NR	Alive, 8 months after surgery	[37]
3	5-FU	Adj	Intravenously	NR	Alive, 15 months after surgery	[21]
4	Capecitabine	Adj	Oral	NR	Alive, 15 months after surgery	[46]
5	Sorafenib	Adj	Oral	NR	Alive, 7 months after surgery	[46]
6	Capecitabine	Adj	Oral	R	Dead	[46]
7	contents unknown	Adj	n.a	R	Dead, 8 months after surgery	[19]
8	contents unknown	Chemo	n.a	PD	Dead	[20]
9	contents unknown	Chemo	n.a	PD	Dead	[46]
10	contents unknown	Chemo	n.a	PD	Dead	[46]
11	contents unknown	Chemo	n.a	PD	Dead	[46]
12	5-FU + CDDP	Chemo	TAI	PD	Dead, 14 months after diagnosis	[45]
13	5-FU + CDDP	Chemo	TAI	PD	Dead, 13 months after diagnosis	[36]
14	GEM + CDDP	Chemo	Intravenously	PD	Dead	[39]
15	GEM + S-1	Chemo	Intravenously	PD	Dead, 10 months after surgery	[17]
16	5-FU + CDDP	Chemo	TAI	PR	Alive, 8 months after chemo	[28]
17	5-FU + CDDP	Chemo	TAI	PR	Alive, 8 months after chemo	[29]
18	GEM + CDDP	Chemo	Intravenously	PR	Alive, 11 months after surgery	[41]
19	S-1 + CDDP	Conversion therapy	TAI	PR	Alive, 6 months after surgery	Our case

Adj, Adjuvant chemotherapy; Cx, Chemotherapy; CDDP, cisplatin; GEM, gemcitabine; n.a., not applicable; No., number; NR, no recurrence; PD, progressive disease; PR, partial remission; R, recurrence; Ref, Reference; S-1, Tegafur/Gimeracil/Oteracil; TAI, transcatheter hepatic arterial injection; 5-FU, 5-fluorouracil

In conclusion, we report a valuable case study of a patient successfully treated with a combination of chemotherapy and conversion surgery for primary hepatic ASC, which is known to have a poor prognosis. Although further number of patients need to be treated to better understand the effectiveness of this treatment strategy, we strongly believe that our study shall serve as a guiding light for similar cases in future.

## Data Availability

All data generated or analyzed during this study are included in this article.

## Abbreviations

ASC: Adenosquamous carcinoma
CDDP: Cisplatin
CT: Computed tomography
FDG/PET: Fluorodeoxyglucose/positron emission tomography
PR: Partial remission
SCC: Squamous cell carcinoma
TAI: Transcatheter hepatic arterial injection
S-1: Tegafur/Gimeracil/Oteracil
5-FU: 5-Fluorouracil
